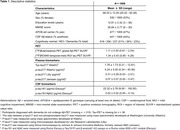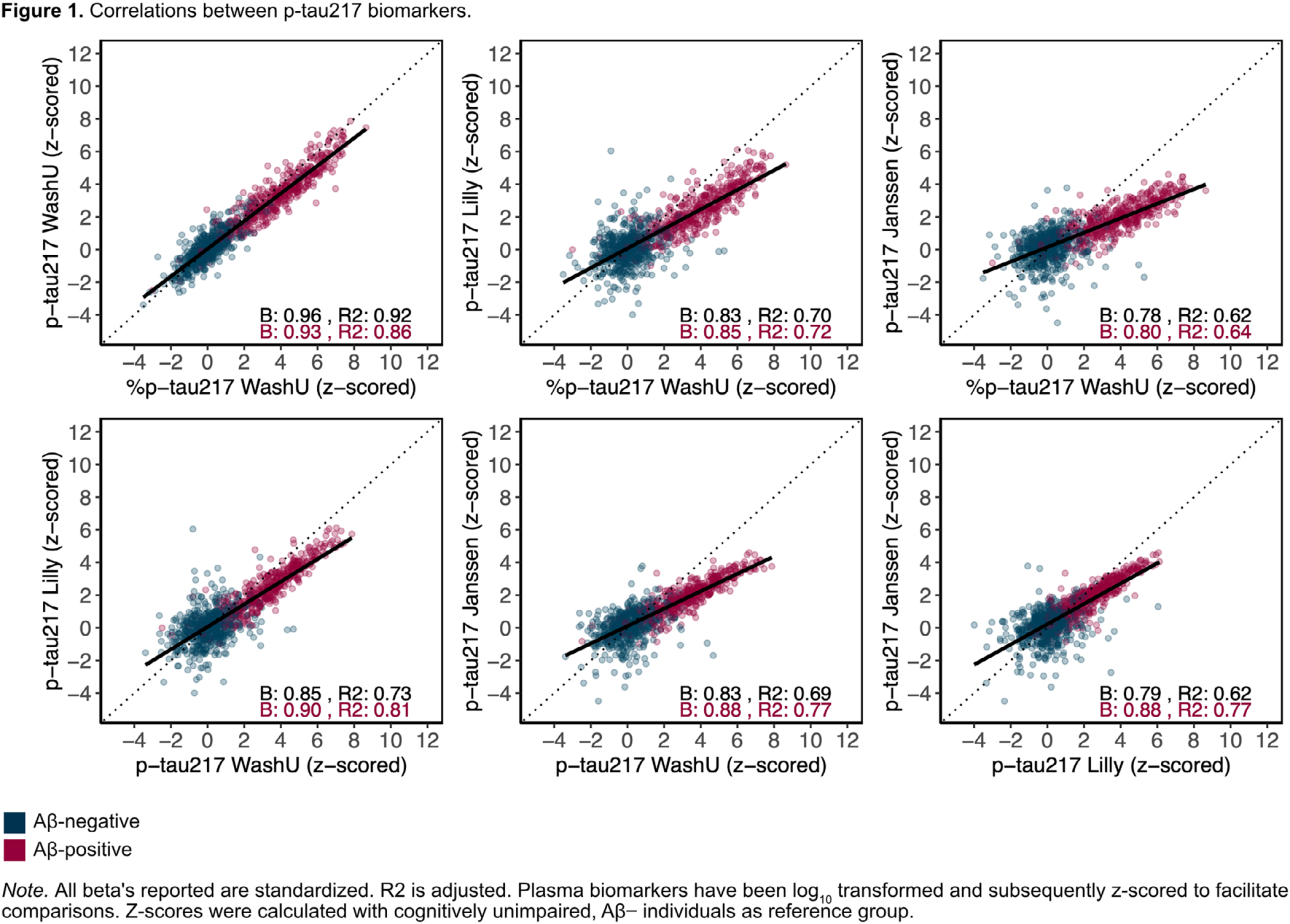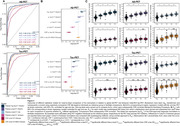# Head‐to‐Head Comparison of Four Plasma Phosphorylated Tau 217 Biomarkers

**DOI:** 10.1002/alz.090618

**Published:** 2025-01-09

**Authors:** Noëlle Warmenhoven, Gemma Salvadó, Shorena Janelidze, Niklas Mattsson‐Carlgren, Hartmuth C. Kolb, Gallen Triana‐Baltzer, Nicolas R. Barthélemy, Randall J. Bateman, Alexa Pichet Binette, Oskar Hansson

**Affiliations:** ^1^ Clinical Memory Research Unit, Department of Clinical Sciences, Lund University, Lund Sweden; ^2^ Skåne University Hospital, Lund Sweden; ^3^ Wallenberg Center for Molecular Medicine, Lund University, Lund Sweden; ^4^ Neuroscience Biomarkers, Johnson and Johnson Innovative Medicine, San Diego, CA USA; ^5^ Johnson and Johnson Innovative Medicine, San Diego, CA USA; ^6^ The Tracy Family SILQ Center, St. Louis, MO USA; ^7^ Washington University School of Medicine, St. Louis, MO USA; ^8^ The Tracy Family SILQ Center, Washington University School of Medicine, St. Louis, MO USA; ^9^ Hope Center for Neurological Disorders, Washington University School of Medicine, St. Louis, MO USA; ^10^ Knight Alzheimer Disease Research Center, St. Louis, MO USA; ^11^ Memory Clinic, Skåne University Hospital, Malmö Sweden; ^12^ Skåne University Hospital, Malmö, 21428 Skåne Sweden

## Abstract

**Background:**

We assessed the efficacy of four plasma phospho‐tau217 (p‐tau217) biomarkers in a head‐to‐head comparison, and against two clinically available CSF biomarkers for Alzheimer’s disease (AD).

**Method:**

Samples were analyzed from 1009 individuals from the Swedish BioFINDER‐2 cohort (Table 1). We included the following biomarkers: %p‐tau217_WashU_, p‐tau217_WashU_ (both mass‐spectrometry), p‐tau217_Lilly_, p‐tau217_Janssen_ (both immunoassays), CSF p‐tau181 and p‐tau181/Aβ42 (Elecsys). Biomarker correlations were assessed using linear regression models. Their discriminative accuracy for global Aβ‐ and temporal meta‐ROI tau‐PET status was evaluated with receiver operating characteristic (ROC) curves. Area under the curve (AUC) values from two ROC curves were compared with DeLong tests. Linear regression models with continuous Aβ‐ and tau‐PET measures were performed. Participants were grouped into PET‐positive quartiles, which were compared with t‐tests. Effect sizes (Cohen’s D (CD)) were calculated between PET‐positive/negative groups, and between neighboring quantiles.

**Result:**

All plasma biomarkers were correlated (0.62≥R_adj_^2^≥0.92, Figure 1). %p‐tau217_WashU_ showed the significantly largest effect size for both Aβ‐PET status and tau‐PET status (CD_Aβ‐PET_=1.635_;_ CD_Tau‐PET_=1.828) compared to the other biomarkers (all p_FDR_ <0.05). p‐tau217_Janssen_ had a lower plasma effect size (CD_Aβ‐PET_=1.313; CD_Tau‐PET_=1.590), but not significantly different from p‐tau217_Lilly_. Although all plasma biomarkers showed high AUCs (0.90‐0.95) for Aβ‐PET positivity, %p‐tau217_WashU_ was the highest, performing significantly better than all other biomarkers including CSF p‐tau181/Aβ42_Elecsys_ (all p_FDR_<0.01) (Figure 2A). A similar pattern was observed for tau‐PET where %p‐tau217_WashU_ also performed significantly better than all other biomarkers except for p‐tau217_WashU_ (all p_FDR<_0.01) (Figure 2A). With continuous PET measures, %p‐tau217_WashU_ showed the highest R_adj_^2^ compared to the other biomarkers for Aβ‐PET and tau‐PET (Figure 2B). In this context, all plasma ptau217 markers performed better that CSF ptau181_Elecsys_. Compared to CSF p‐tau181/Aβ42_Elecsys_, p‐tau217_Lilly_ and p‐tau217_WashU_ performed similarly whereas %p‐tau217_WashU_ performed significantly better. Quantile grouping revealed that all biomarkers showed significant differences when distinguishing between negatives and early‐stage positives for both Aβ‐PET and tau‐PET, with %p‐tau217_WashU_ consistently having the significantly largest effect size (Figure 2C). For tau‐PET, plasma biomarkers distinguished better between disease stages compared to CSF.

**Conclusion:**

When predicting Aβ‐ and tau‐PET load, both mass‐spectrometry and immunoassay methods detecting plasma p‐tau217 perform similarly to an FDA‐approved CSF test, with %p‐tau217_WashU_ performing even better.